# Growth of for‐profit involvement in emergency medicine graduate medical education and association between for‐profit affiliation and resident salary

**DOI:** 10.1002/aet2.10786

**Published:** 2022-08-03

**Authors:** Jared W. Lassner, James Ahn, Armaan Singh, Paul Kukulski

**Affiliations:** ^1^ University of Chicago Pritzker School of Medicine Chicago Illinois USA; ^2^ University of Chicago Medical Center, Section of Emergency Medicine Chicago Illinois USA

**Keywords:** EM physician oversupply, EM residency growth, for‐profit hospitals, graduate medical education, HCA Hospitals, resident salary

## Abstract

**Background:**

Following projections of an emergency medicine (EM) physician oversupply, the growth of EM residency programs affiliated with for‐profit hospitals has been subject to increased attention and speculation. However, essentially no literature exists regarding these programs. Resident pay is one area where these programs could differ from nonprofit‐affiliated programs, as investor obligations could make for‐profit corporations more likely to reduce resident salaries to increase profit margins. Here, we aim to quantify the growth of EM for‐profit affiliated residency programs from 2001–2021 and determine if PGY1 salaries differ between these program types.

**Methods:**

Medicare and ACGME accreditation data were used to determine the profit status of hospitals affiliated with EM residency programs. ACGME new accreditation data from 2001–2021 were used to quantify the growth of both for‐profit and nonprofit affiliated programs over this period. We searched program websites and called programs to determine 2021–2022 PGY1 salary. Multiple regression was used to model the relationship between profit status and salary using program characteristic covariates to control for confounding variables.

**Results:**

The number of EM programs increased from 117 to 276 from 2001–2021 while the number of for‐profit affiliated EM residency programs increased from 1 to 29 during this period. Most (85.7%, [24/29]) for‐profit affiliated programs were accredited from 2016–2021. Mean for‐profit affiliated program salary ($55,658, *n*  = 24) was $3840 lower than mean nonprofit affiliated program salary ($59,498, *n*  = 203). For‐profit affiliation was a significant predictor of lower 2021–2022 PGY1 salary after controlling for other program characteristics using multiple regression ( *ß*  = −1919.88, P = 0.010).

**Conclusions:**

We found a substantial growth of newly ACGME accredited for‐profit affiliated EM residency programs from 2016–2021. We also found for‐profit affiliated programs pay lower PGY1 salaries than nonprofit–affiliated programs after controlling for potential confounding variables, which suggests more oversight over the salary determination process could be necessary to prevent resident underpayment.

## INTRODUCTION

For‐profit corporation involvement in emergency medicine (EM) graduate medical education (GME) has been the subject of increased attention and speculation following a workforce study that projected a surplus of nearly 8000 EM physicians by 2030.^1^ Some have suggested that the proliferation of residency programs affiliated with for‐profit hospitals could be contributing to this oversupply.^2^ While both the extent of growth and the intentions of for‐profit hospitals in EM GME have been questioned,^2^, ^3^ meaningful differences between for‐profit and nonprofit hospitals are not always apparent, as some studies have failed to find evidence that nonprofit hospitals are more likely to serve public interests via increased charity care.^4^, ^5^ Despite the uncertainty surrounding the role of for‐profit hospitals in GME, currently almost no literature exists regarding these for‐profit affiliated EM residency programs, and little is known about the extent of their growth or potential differences between for‐profit and nonprofit affiliated EM residency programs.

One area that may differ between these program types is resident salary—a topic attracting increased attention following resident calls for unionization at several institutions.^6^, ^7^ A 2021 survey found that the majority of resident physicians across all specialties are dissatisfied with their salary.^8^ The same survey found that the average PGY1 salary was $57,500, which, conservatively estimating a 60 hour work week (44% of residents reported spending more than 60 hours in the hospital per week), represents an hourly compensation of less than $20.^8^ In comparison, the median salary of all 25‐ to 34‐year‐old full‐time workers with a master's or higher degree was found to be $69,700 in 2020.^9^ First‐year residents will also typically have at least 8 years of post‐secondary education and carry an average debt of $200,000,^10^ which likely contribute to the perception that their pay level is inadequate.

There are several factors that reinforce the current level of resident pay, including a match process that insulates programs from market forces that traditionally influence salary levels and an opaque salary determination process with limited oversight. The main funding source for resident salaries is Medicare Direct Graduate Medical Education (DGME) payments, which are calculated by multiplying a base period per‐resident amount by the number of full‐time equivalent residents and the Medicare share of total inpatient days.^11^ Hospitals have significant autonomy in allocating this funding and determining resident salary schedules. Both nonprofit and for‐profit affiliated programs may be incentivized to leverage the opaque determination process, lack of oversight, and freedom from typical market forces on salary by reducing resident pay to increase profit margins. However, due to the additional financial pressure for‐profit hospitals face from investors, we hypothesize that these institutions could be particularly motivated to increase profit margins by reducing resident pay, potentially creating a salary disparity between for‐profit and nonprofit‐affiliated EM residency programs. In this study, we aim to calculate the growth of for‐profit and nonprofit‐affiliated residency programs in EM and analyze the relationship between profit affiliation and resident salary.

## METHODS

### Data collection

The method for classifying programs as nonprofit or for‐profit affiliated was adopted from our prior study comparing board certifying exam pass rates between these program types.^12^

A list of all accredited EM residency programs as of August 2021 was acquired from the public ACGME accreditation database, along with the name and date of accreditation of every program accredited from 2001–2021.^13^ We then determined the primary affiliated clinical site. If the name of the residency program included a single hospital or clinic, this location was used as the primary affiliated site. If no sites or multiple sites were included in the name, Doximity residency navigator was used to determine the site where the largest number of months are spent. After determining this primary site, Centers for Medicare & Medicaid Services (CMS) data were used to classify these hospitals as for‐profit or nonprofit.^14^ We searched the internet to confirm current for‐profit hospital status and determine date of acquisition for hospitals that CMS indicated to be under proprietary control; we also determined the corporate affiliates of for‐profit affiliated programs using individual corporation websites to confirm specific corporate ownership. If CMS indicated a hospital to be nonprofit, we used internet searches to confirm current nonprofit status and determine if nonprofit‐affiliated programs had been previously for‐profit affiliated at any point. Programs were included as for‐profit affiliated for the purposes of this study if the affiliated hospital held for‐profit status at the time data were collected in August 2021. See Figure S1 in the supplemental content for further details regarding profit status determinations.

We searched residency program websites to determine 2021–2022 PGY1 salary. If no EM specific salary was found on the website, current salary information from other specialties at the institution was used. If no current salary was listed for any specialty, we called programs using phone numbers available on public program websites to obtain this information. Graduate medical education phone numbers were prioritized if available, followed by EM program numbers and finally other specialty programs if the former two could not be reached. If another specialty program was used to determine salary, it was always confirmed that salaries are identical across PGY levels at the institution. See Figure S2 in the supplemental content for further details regarding salary determinations.

### Data analysis

The ACGME accreditation data and profit status determinations were used to quantity the growth of new for‐profit affiliated programs from 2001–2021. We determined the number of programs that became accredited each year and determined if each program was for‐profit affiliated or nonprofit affiliated to calculate growth in the number of each program type throughout the 2001–2021 period. Public data acquired from osteopathic.org were used to determine the programs that had previously held American Osteopathic Association (AOA) accreditation and recalculate new program growth after removing these programs. We also determined the geographic distribution of for‐profit affiliated residency programs. Programs no longer accredited as of August 2021 were not included in these analyses.

We used multiple linear regression to analyze the relationship between profit status and salary and included the following covariates to control for confounding variables: program size, cost of living, year founded, census region, program type, Medicare share of inpatient days, and estimated DGME funding per resident. These variables were chosen because they were publicly available, and we hypothesized these variables could reasonably influence the relationship between profit status and resident salary. Program size and year accredited (if prior to 2001) were acquired from Doximity residency navigator. To estimate cost of living we used 2019 Census Bureau Regional Price Parities,^15^ a measure used in a prior study analyzing resident salaries.^16^ Four programs were located in areas with no metropolitan statistical area designation and therefore the cost of living estimate was not included for these four programs. Program type (community based, university based, community based; university affiliated, or military based) was acquired from the American Medical Association's Fellowship and Residency Electronic Interactive Database (AMA FREIDA). 2019 Medicare cost reports were used to calculate Medicare share of inpatient days (Medicare Inpatient Days/Total Inpatient Days) and estimate DGME funding per resident (GME Part A+ Part B/total interns and residents). Military and Puerto Rico based programs were excluded from our analyses. This study was deemed exempt from institutional review by the University of Chicago IRB. P < 0.05 was considered significant. SPSS version 28 was used for analyses.

## RESULTS

**FIGURE 1:**
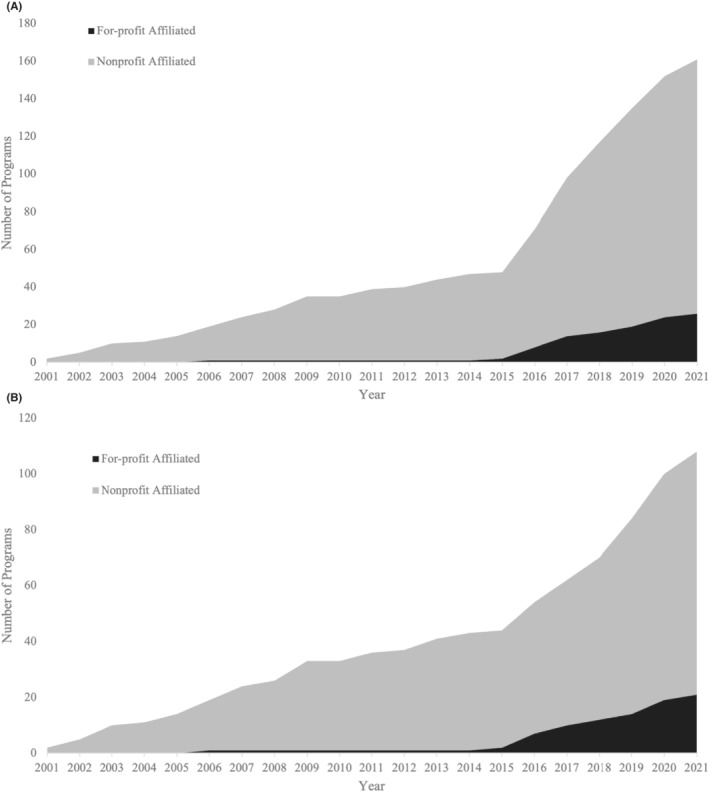
(A) Cumulative number and affiliations of programs accredited during the 2001–2021 period. (B) Cumulative number and affiliations of programs accredited during the 2001–2021 period after removing former osteopathic programs.

**FIGURE 2:**
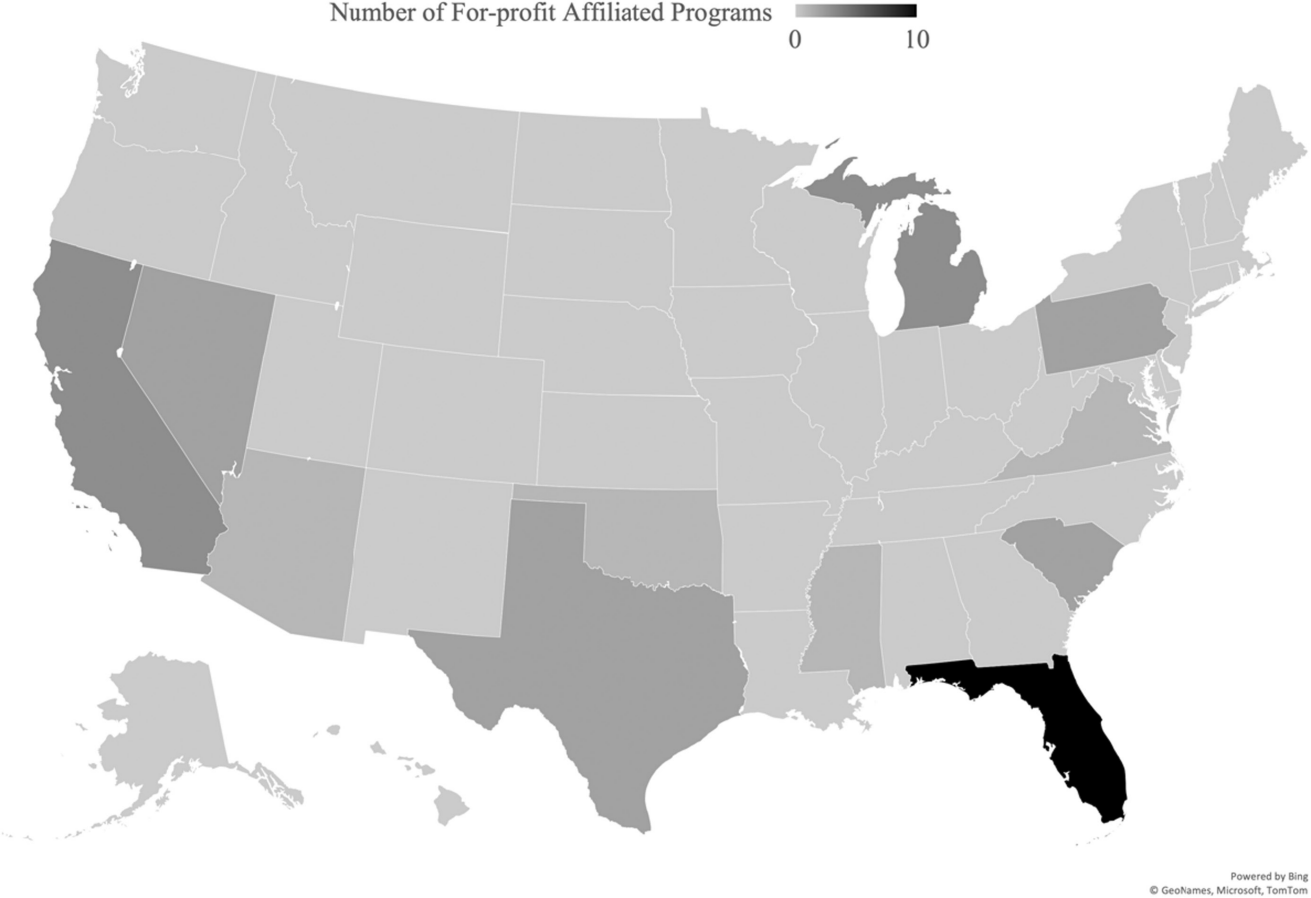
Geographic distribution of for‐profit affiliated programs.

During the 2001–2021 period, the number of EM residency programs increased from 117 to 276, which corresponds to a 135.9% increase. During this time the proportion of for‐profit affiliated EM residency programs increased from 1/117 to 29/276, corresponding to a 1129.4% increase. Most (85.7%, 24/29) of the for‐profit affiliated programs were accredited between 2016–2021. The number of for‐profit affiliated and nonprofit affiliated programs accredited during the 2001–2021 period is shown in Figure 1A and B. The geographical distribution of for‐profit affiliated programs as of 2021 is shown in Figure 2. The most prevalent corporate entity affiliated with programs was HCA Healthcare (58.6%) (17/29), followed by Tenet Healthcare (13.8%) (4/29).

We were able to obtain salary data for 70.3% (189/269) of eligible programs simply by searching program websites. Of the remaining programs we were able to obtain 47.5% (38/80) of salaries by calling programs. In total, we were able to obtain 2021–2022 salaries for 84.4% (227/269) of eligible programs. The rate of salary data acquisition was similar between for‐profit affiliated programs (82.8%, [24/29]) and nonprofit‐affiliated programs (84.6%, [203/240]).

**FIGURE 3:**
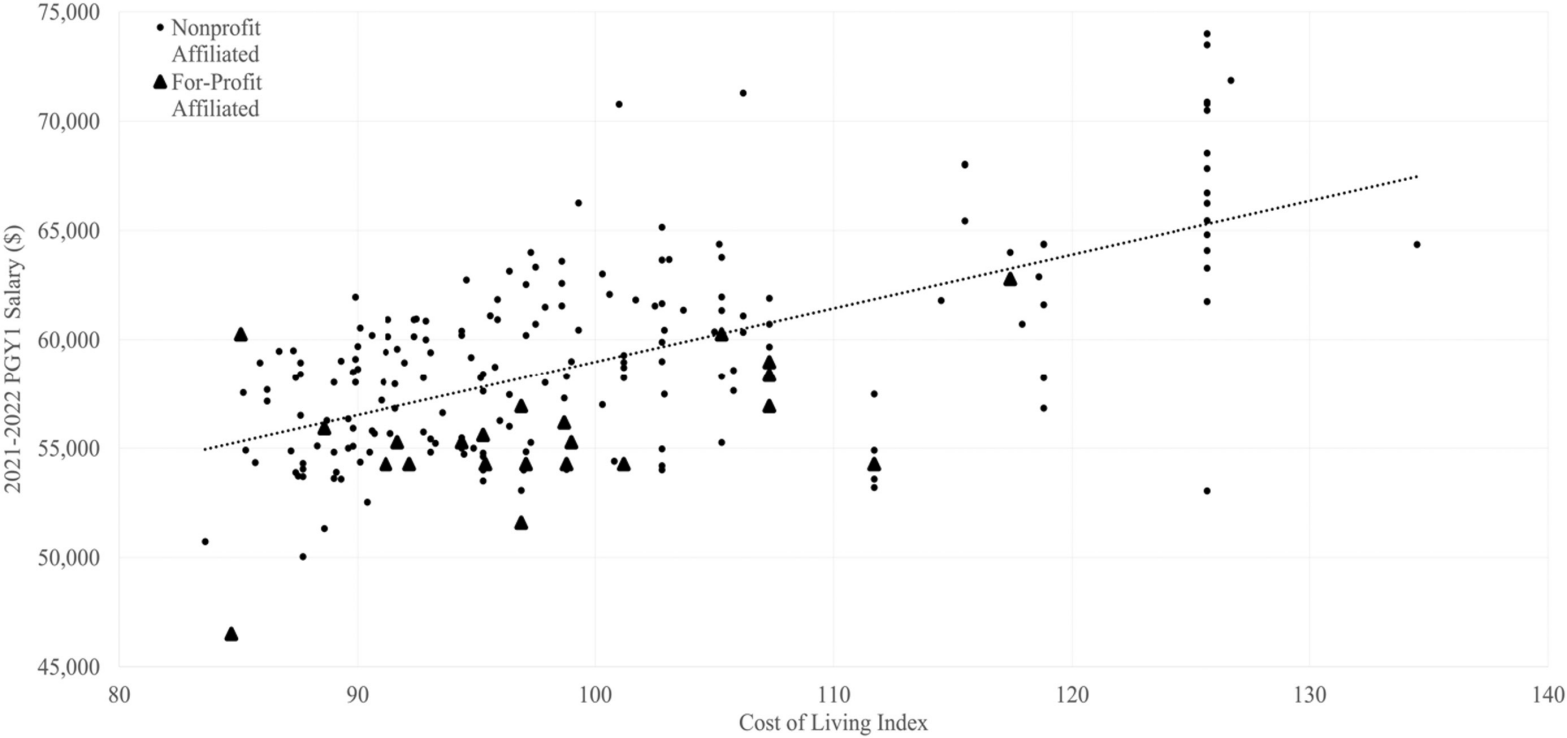
Relationship between cost of living index and 2021–2022 PGY1 salary stratified by program profit status affiliation.

Mean for‐profit affiliated program salary ($55,658, *n* = 24) was $3840 lower than mean nonprofit affiliated salary ($59,498, *n* = 203). Figure 3 shows the relationship between cost of living and salary stratified by profit status. Multiple regression revealed that for‐profit affiliation was a significant predictor of lower 2021–2022 PGY1 salary after controlling for program size, cost of living, year founded, census region, program type, Medicare share of inpatient days, and estimated DGME funding per resident (*ß* = −1919.88, P = 0.010). The adjusted R^2^ for the model was 0.597. Complete regression results are shown in Table S1 in the supplemental material.

## DISCUSSION

There has been a considerable proliferation of for‐profit affiliated EM residency programs, specifically during the 2016–2021 period and led by HCA Healthcare, which supports the growing notion that there is an increasing presence of for‐profit corporations in EM GME. This pattern may not be unique to EM, as our prior work has shown a similar growth pattern of for‐profit affiliated programs in the fields of internal medicine and general surgery.^12^ The stark increase in total programs accredited after 2015 can be partially explained by former AOA programs becoming ACGME accredited prior to the residency merger; however, Figure 1B shows that even when these former AOA programs are removed from the analysis there remains a substantial increase in total newly accredited programs during the 2016–2021 period. The geographic distribution of all for‐profit affiliated programs (Figure 2) reveals that for‐profit affiliated programs are mostly located in southern states and supports the previously suggested idea that Florida is producing many for‐profit affiliated programs.^17^

The profitability of operating residency programs is almost entirely unknown and likely varies significantly across different specialties and program charactersitics.^18^ However, assuming that for‐profit institutions are unlikely to intentionally pursue unprofitable ventures,^19^, ^20^ the recent increase in EM residency programs sponsored by for‐profit hospitals implies that operating EM GME programs is likely profitable. If this is the case, EM residents are providing excess value to hospital systems relative to their cost. It is important to note that profit is not a motive unique to for‐profit corporations; nonprofit‐affiliated programs likely similarly enjoy direct profit from these programs, which, in combination with the other indirect benefits of training residents such as increased prestige and access to a constant stream of new physicians to hire,^21^, ^22^ could potentially contribute to the recent massive growth of both nonprofit and for‐profit affiliated programs.

Despite this potential shared motive, we found for‐profit affiliated programs tended to pay lower PGY1 salaries even when accounting for potential confounding variables, which suggests that resident physicians at these programs could be particularly underpaid relative to their peers at nonprofit affiliated programs. This could especially impact residents with low‐income backgrounds, large debt burdens, or those with children or planning to have children during residency. Medical students belonging to these groups should be advised to consider salary when ranking programs, as we have identified considerable salary variability across EM programs, as well as a difference in salary based on profit affiliation type. This latter discrepancy can best be visualized in Figure 3, where almost every for‐profit affiliated program falls below the line of best fit when plotting salary by cost of living. We suggest that this difference may be attributed to investor obligations: although both for‐profit and nonprofit‐affiliated programs likely profit from residency programs, investor obligations might place additional pressure on for‐profit corporations to maximize profit margins.

Based on these findings we recommend that more oversight over the salary determination process by external bodies is necessary to prevent resident underpayment, as the unique nature of the match limits typical market regulation of salaries and incentivizes hospitals to underpay. Not only would this protect residents from underpayment, but this would also reduce the profit margins for operating residency programs, which could disincentivize program proliferation and potentially slow the rate of EM physician growth.

Another potential solution to quell the rapid growth of programs identified here is the formation of a body specific to EM tasked with regulating the number of programs achieving accreditation based on current specialty needs. While the ACGME oversees new program accreditation, the organization acts by accrediting programs that meet its accreditation standards, rather than selectively approving programs based on specialty specific market needs.^23^ The potential for new residency programs is therefore only limited by the number of hospitals that meet the minimum requirements. The creation of the aforementioned entity could potentially inhibit the excess proliferation of programs in a climate of resident oversupply.

Future work is needed to understand why for‐profit affiliated programs are rapidly expanding in EM, as well as to what degree their growth contributes to the recently described projected oversupply of EM physicians. Follow‐up studies should utilize survey methods to determine if the salary discrepancy identified here impacts residents' quality of life, as well as further explore additional differences that could exist between nonprofit and for‐profit affiliated programs. Studies should also examine the role and impact of contract management groups (CMGs) in clinical sites involved in EM GME. Many emergency rooms in the United States are staffed by large corporate‐owned CMGs.^24^ CMG staffing is independent of the overall profit status of the hospital; therefore, even in nonprofit affiliated programs, corporate interests could be impacting residency experiences via CMG influence.

## LIMITATIONS

Our study is limited by our sole focus on salaries without consideration for benefits. Programs can vary widely regarding specific benefits they offer, which can include technology, food, travel allowances, paid time off, childcare, etc. These benefits can significantly alter the total compensation package at a given program and could compensate for a lower salary; thus, exclusion of these factors is a major limitation of our data.

There are also limitations to the sources we used to collect the data. We relied heavily on public sources like Doximity residency navigator and internet searches, and it is possible that these sources provided incorrect information. While we attempted to determine profit status in an unbiased and systematic way, it is possible that status could have been misattributed in some instances due to human error or hospital affiliation changes. Further, we were unable to acquire salary information for all programs. It is also possible that program representatives contacted via phone calls provided incorrect or outdated salary information.

Additionally, there are potential market changes that we did not account for that could impact the accuracy of our data. The newly accredited program analyses used to create Figure 1 assumed that current hospital profit status applies to programs when they were accredited, but this is not always the case as changes in profit status due to acquisitions do occur. However, the number of programs affiliated with hospitals that changed profit status over this time was found to be small and likely would not impact the trends we identified. We also did not include programs with withdrawn accreditation as of 2021 in our analyses. This likely slightly lowers the number of programs we found to be accredited during this time from the true value and implies that our calculation for the number of programs accredited at the start of the 20‐year period in 2001 is likely an underestimate.

Finally, it is important to note that in this study we only examined corporate involvement in GME through the profit status of hospitals affiliated with residency programs. We did not consider the involvement of private‐equity‐backed CMGs in GME, which is another avenue through which for‐profit corporations could be involved in GME.

## CONCLUSIONS

The number of ACGME accredited EM residency programs affiliated with for‐profit hospitals has increased considerably over the past 5 years. These programs tend to pay their residents lower salaries, even after controlling for other program factors that could influence salary. We suggest that more oversight over the salary determination process could be necessary to ensure salary equity across all program types to not only protect residents from underpayment, but also to potentially disincentivize the excess production of new programs. Future work is needed to explore reasons for the proliferation of for‐profit affiliated EM residency programs and other differences that could exist between these program types.

## AUTHOR CONTRIBUTIONS

JL was responsible for the study concept and design. JL and AS were responsible for acquisition of the data. JL, JA, AS, and PK contributed to the analysis and interpretation of the data. JL was responsible for drafting of the manuscript. JL, JA, AS, and PK were responsible for critical revision of the manuscript for important intellectual content. JL takes responsibility for the paper as a whole.

This work was presented at SAEM 2022.

## CONFLICT OF INTEREST

This study was not funded. We have no conflicts of interest to report.

## Supporting information


Figure S1

Figure S2

Table S1
