# Supplementary figures and images for: Growth of for‐profit involvement in emergency medicine graduate medical education and association between for‐profit affiliation and resident salary
Source: AEM Educ Train. 2022 Aug 3;6(4):e10786. doi: 10.1002/aet2.10786 (PMC9348842; doi:10.1002/aet2.10786)

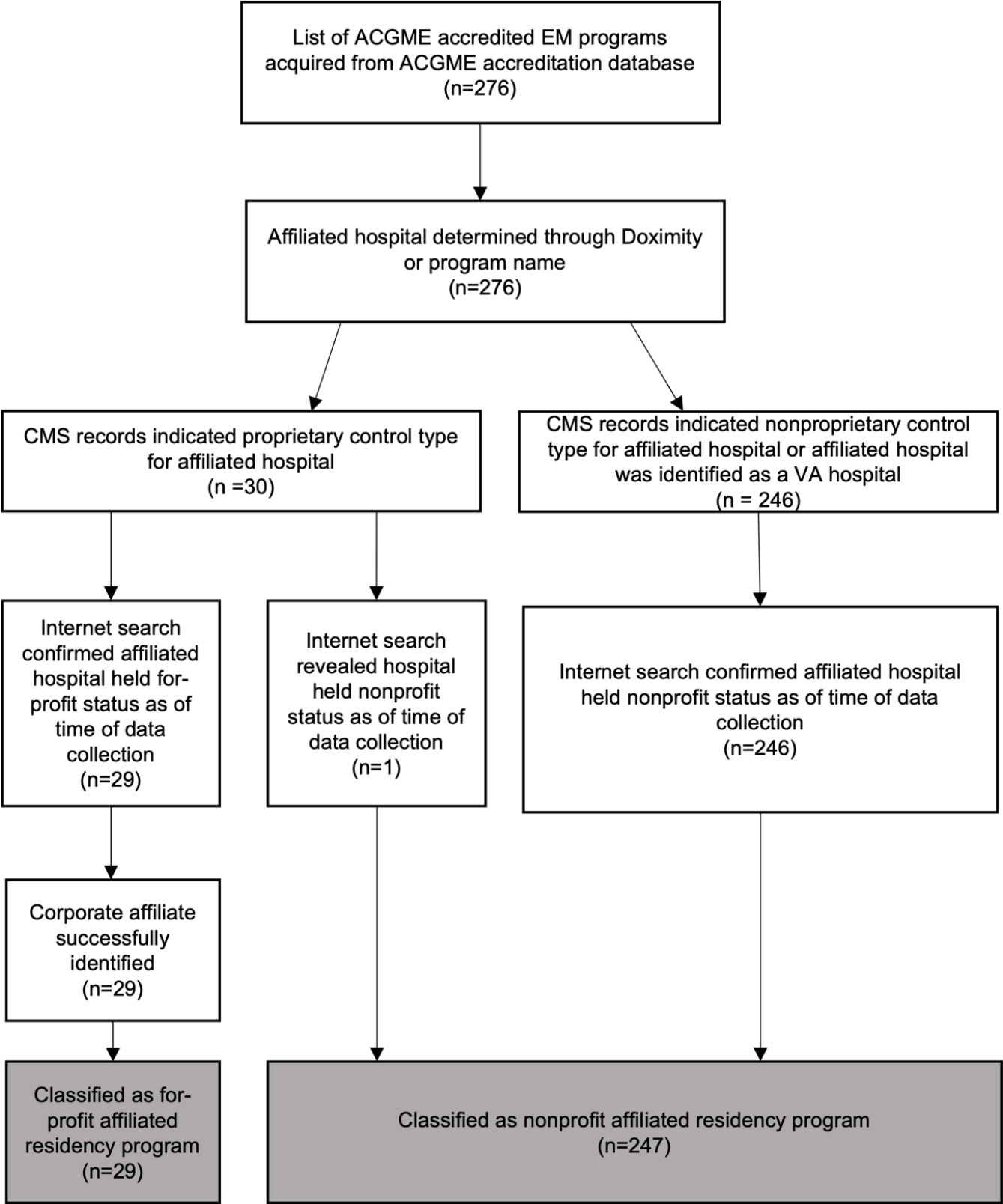

Supplement: Supplementary file 1 — Figure S1 [file AET2-6-e10786-s001.pdf]

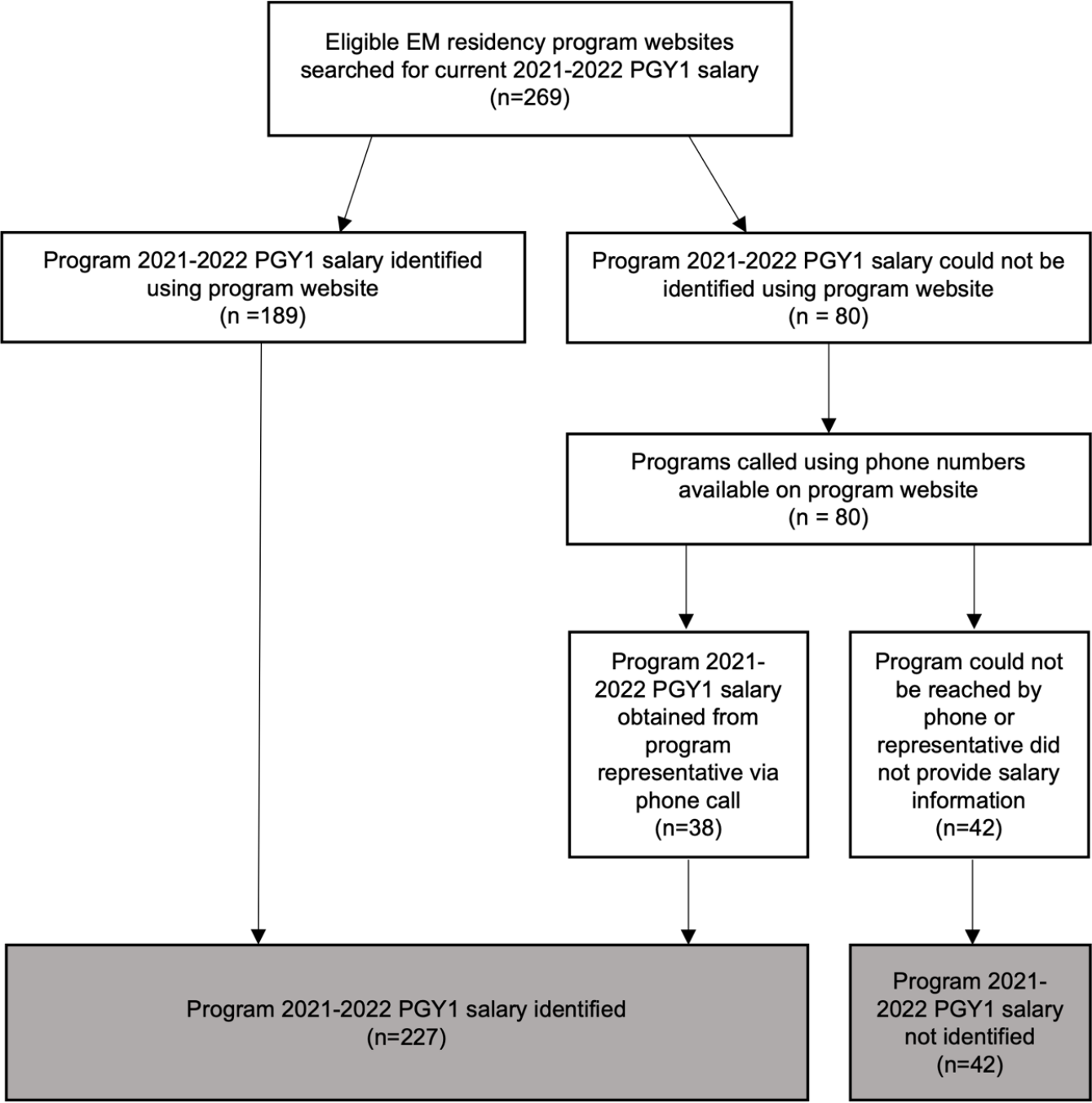

Supplement: Supplementary file 2 — Figure S2 [file AET2-6-e10786-s003.pdf]
