# Supplementary material for: Growth of for‐profit involvement in emergency medicine graduate medical education and association between for‐profit affiliation and resident salary
Source: AEM Educ Train. 2022 Aug 3;6(4):e10786. doi: 10.1002/aet2.10786 (PMC9348842; doi:10.1002/aet2.10786)
Supplement: Supplementary file 3 — Table S1 [file AET2-6-e10786-s002.docx]

**Supplemental Material**

**Table S1: Complete linear regression results.**

| Program Characteristic | Unstandardized ß value (Standard Error) | P Value |
| --- | --- | --- |
| For-Profit Affiliated | -1919.88 (737.91) | 0.010 |
| Cost of Living Index | 168.57 (22.31) | <0.001 |
| Size | 49.71 (18.77) | 0.009 |
| Year Founded | -1.70 (19.96) | 0.932 |
| West | 333.47 (764.60) | 0.663 |
| South | -1787.90 (574.37) | 0.002 |
| Northeast | 2390.92 (670.15) | <0.001 |
| Midwest |  | REF |
| Community Based; University Affiliated |  | REF |
| Community Based | -332.74 (648.44) | 0.608 |
| University Based | 1522.18 (501.22) | 0.003 |
| Medicare share of total inpatient days | 60.61 (31.58) | 0.056 |
| Estimated DGME Funding per resident | 0.017 (0.018) | 0.348 |

Midwest and Community Based; University Affiliated programs were excluded as reference variables for location and program type. Adjusted R^2^ for this model was 0.597.
